# Novel genetic markers for chronic kidney disease in a geographically isolated population of Indigenous Australians: Individual and multiple phenotype genome-wide association study

**DOI:** 10.1186/s13073-024-01299-3

**Published:** 2024-02-12

**Authors:** Vignesh Arunachalam, Rodney Lea, Wendy Hoy, Simon Lee, Susan Mott, Judith Savige, John D. Mathews, Brendan J. McMorran, Shivashankar H. Nagaraj

**Affiliations:** 1 Centre for Genomics and Personalised Health and School of Biomedical Sciences, Queensland University of Technology, Brisbane, QLD Australia; 2 Centre of chronic disease, Faculty of Medicine, University of Queensland, Brisbane, QLD Australia; 3 Royal Melbourne Hospital, The University of Melbourne, Melbourne, VIC Australia; 4 Melbourne School of Population and Global Health, The University of Melbourne, Melbourne, VIC Australia; 5 National Centre for Indigenous Genomics, The John Curtin of Medical Research, Australian National University, Canberra, ACT Australia; 6 Translational Research Institute, Queensland University of Technology, Brisbane, QLD Australia

**Keywords:** Tiwi Island, Chronic Kidney Disease, Albumin-to-creatinine ratio, Genetic variant, Indigenous Australians

## Abstract

**Background:**

Chronic kidney disease (CKD) is highly prevalent among Indigenous Australians, especially those in remote regions. The Tiwi population has been isolated from mainland Australia for millennia and exhibits unique genetic characteristics that distinguish them from other Indigenous and non-Indigenous populations. Notably, the rate of end-stage renal disease is up to 20 times greater in this population compared to non-Indigenous populations. Despite the identification of numerous genetic loci associated with kidney disease through GWAS, the Indigenous population such as Tiwi remains severely underrepresented and the increased prevalence of CKD in this population may be due to unique disease-causing alleles/genes.

**Methods:**

We used albumin-to-creatinine ratio (ACR) and estimated glomerular filtration rate (eGFR) to estimate the prevalence of kidney disease in the Tiwi population (*N* = 492) in comparison to the UK Biobank (UKBB) (*N* = 134,724) database. We then performed an exploratory factor analysis to identify correlations among 10 CKD-related phenotypes and identify new multi-phenotype factors. We subsequently conducted a genome-wide association study (GWAS) on all single and multiple phenotype factors using mixed linear regression models, adjusted for age, sex, population stratification, and genetic relatedness between individuals.

**Results:**

Based on ACR, 20.3% of the population was at severely increased risk of CKD progression and showed elevated levels of ACR compared to the UKBB population independent of HbA1c. A GWAS of ACR revealed novel association loci in the genes *MEG3* (chr14:100812018:T:A), *RAB36* (rs11704318), and *TIAM2* (rs9689640). Additionally, multiple phenotypes GWAS of ACR, eGFR, urine albumin, and serum creatinine identified a novel variant that mapped to the gene *MEIS2* (chr15:37218869:A:G). Most of the identified variants were found to be either absent or rare in the UKBB population.

**Conclusions:**

Our study highlights the Tiwi population’s predisposition towards elevated ACR, and the collection of novel genetic variants associated with kidney function. These associations may prove valuable in the early diagnosis and treatment of renal disease in this underrepresented population. Additionally, further research is needed to comprehensively validate the functions of the identified variants/genes.

**Supplementary Information:**

The online version contains supplementary material available at 10.1186/s13073-024-01299-3.

## Background

The quest for the genetic basis of chronic diseases has led to the discovery of several genes and loci. Approaches such as genome-wide association studies (GWASs) established significant associations between genomic variants and complex traits at the population level with the potential to inform diagnosis, preventative health management, personalized therapy, and clinical outcomes [1, 2]. However, there is a notable “genomic divide” between Indigenous and non-Indigenous populations [3], as those who endure the highest burden of chronic diseases benefit the least from advancements in genetic research [4]. Indigenous representation in genome-wide association studies (GWAS) was estimated at 0.05% in 2016 [5] and 0.02% in 2019 [6]. Indigenous populations are also conspicuously absent from reference variant data and not represented in the biobank such as the Genome Aggregation Database (gnomAD) and the UK biobank (UKBB), which include global data from 138,632 and 450,000 individuals, respectively. Australian Indigenous populations, in particular, are underrepresented in genomic studies, including the 0.05% of Native peoples included in GWAS datasets [5]. Although global efforts such as the Silent Genomes Project (2017) and the Aotearoa Variome Project (2019) aim to create background variant databases (BVD) for Indigenous populations in Canada and New Zealand, respectively, efforts to include Australian Indigenous populations in variant databases are lacking, thereby widening the gap in equitable genomic healthcare between Indigenous and non-Indigenous Australians.

Chronic Kidney Disease (CKD) is defined as the gradual loss of kidney function over a period of time and is diagnosed by an estimated glomerular filtration rate (eGFR) of < 60 ml/min/1.73 m^2^ for ≥ 3 months [7, 8]. It is one of the most prevalent chronic diseases affecting more than 840 million people globally in 2017, thereby impacting ~ 13.4% of the global population [9]. Although CKD is on track to be the 5th leading cause of years of life lost by 2040 [10], kidney disease has not received ample attention [9]. Notably, the prevalence of CKD is considerably higher in Indigenous populations, with a twofold higher rate reported for Indigenous Canadians [11] and 6- and eightfold higher rates of end-stage renal disease and non-dialysis CKD hospitalizations, respectively, for Indigenous compared to non-Indigenous Australians [12]. Moreover, the age of onset for CKD in Indigenous populations is much lower than in other ethnic groups, and when combined with limited access to medical services and treatments such as dialysis and kidney transplantation, results in a higher incidence of premature mortality in these populations [13].

Over the past decade, genetic research using GWASs has revealed more than 600 genes implicated in both monogenic disorders and syndromic diseases that impact the kidney and urinary tract [14]. The largest trans-ancestry GWAS meta-analysis for eGFR, which involved over a million participants, identified 424 loci, 201 of which were novel [15]. A 2019 trans-ethnic GWAS of eGFR in 280,722 individuals, with replication in 765,289 individuals from the Chronic Kidney Disease Genetic (CKDGen) Consortium, identified 136 genome-wide significant loci and 82 novel variants, as well as 36 novel genes via genetically predicted gene expression associated with renal function that were not previously identified by GWAS [16]. In addition to the identification of loci associated with eGFR, GWASs of diverse populations have also identified additional loci associated with urinary ACR [17, 18], and serum urate levels [19]. The Population Architecture using Genomics and Epidemiology (PAGE) study, which investigated CKD-associated variants in ethnic minority communities in the USA, identified a novel variant associated with *NMT2* and evidence of association with *APOL1* [20]. The discovery of *APOL1* is significant in pointing toward population-specific causes of CKD in African Americans [21], who face a higher burden of CKD than Europeans. However, with few exceptions [16, 20, 22], the proportion of GWAS studies and publicly available databases involving underrepresented populations has either plateaued or decreased in the last several years [23]. Notably, the lack of available reference genomes and databases for Indigenous populations also limits our ability to study specific CKD-associated variants that may have novel interactions in these populations, hindering efforts to develop new avenues for early diagnosis and treatment.

The Tiwi Islanders are a genetically distinct Indigenous population—are one of the founder populations of Australian Aboriginal communities and are thought to have remained in relative isolation from mainland peoples since the islands they inhabit became separated from the mainland by the Clarence Straight 7000–15,000 years ago [24, 25]. In a study of renal disease and other co-morbidities in Australian Aboriginals, the Tiwi people exhibited a substantially higher predisposition to CKD compared to other Indigenous populations, which was correlated with both age and waist measurements, while their risk of hypertension or diabetes is comparable to that of other Indigenous groups tested [26]. Additionally, kidney disease—is measured by the urinary albumin-to-creatinine ratio (ACR) which is defined as the ratio of urine albumin-to-urine creatinine. ACR was found to be significantly heritable in the Tiwi individuals, in which a deletion in angiotensin-converting enzyme (*ACE*) and a specific mutation in tumor protein 53 (*p53*) contributed approximately 15% of the total heritability. This study further determined that 64% ACR heritability in the Tiwi population is attributed to six polymorphisms across four candidate genes, highlighting genetic contributions to the elevated presence of chronic disease in this population [27]. Despite this, very few studies to date have used genome-wide approaches to characterizing the genes and loci responsible for CKD in the Tiwi population.

The present study represents the most in-depth phenotypic and genotypic analysis of the genetic determinants of CKD in Indigenous Australians to date. Although one GWAS investigation of CKD in a sample of 249 Tiwi individuals identified eight single nucleotide polymorphisms (SNPs), four of which were found to be significantly associated with ACR upon re-testing [25]. The Affymetrix SNP array 5.0 was used in this study which is more suitable for European and West African populations [28] and failed to capture the full extent of the variants responsible for CKD in the Tiwi cohort owing to its low resolution for this population [25]. Thus, to address these gaps and further elucidate the genetic underpinnings of CKD in the Tiwi people, we utilized clinical and whole-genome sequencing data. Our objectives were three-fold: (1) identify the key markers associated with the kidney disease, stratify CKD prognosis, and assess the risk of kidney disease in the Tiwi cohort using available phenotypes, (2) estimate the heritability and perform a GWAS analysis for the individual quantitative phenotypes, (3) perform a GWAS analysis for multiple phenotypes combined to identify the pleiotropic loci. This current research study is a significant milestone in the field of scientific enquiry to understand the genetic architecture of CKD in the underrepresented population.

## Methods

### Study population and datasets

The whole genome sequence data for this study were acquired from blood samples collected from 492 Tiwi individuals between 2013 and 2014, representing approximately 40% of Tiwi adults [25]. The associated phenotypic profiles include blood pressure, height, weight, waist circumference, glycated hemoglobin, diabetes diagnosis, serum and urine albumin, serum, and urine creatinine, urinary ACR, and eGFR (estimated using CKD-EPI 2021 Eq. [29]). Whole genome sequencing (WGS) of the Tiwi cohort was performed in four batches using Illumina paired-end sequencing (Illumina Novaseq 6000; Illumina, Inc., San Diego, CA, USA) with an average coverage of > 30 × . Both genotypic and phenotypic data were available for only 461 of 492 individuals. The quality control steps were performed using plink v1.9 and include missing genotype rate (–geno 0.02 –mind 0.1), Hardy–Weinberg Equilibrium (*p *> 5*10^–8^), heterozygosity rate (± 3 standard deviation), and minor allele frequency (–maf 0.05). After quality control (QC) steps, samples from 455 individuals and 4.9 million SNPs were utilized for the genotype-to-phenotype association. Subsequently, 150,000 whole genome sequences and corresponding phenotype data from UK Biobank (UKBB) data were used to compare allele frequency and clinical data between the Tiwi and the UKBB population, which included the following subgroups: African (*n* = 1320), British (*n* = 124,948), Caribbean (*n* = 1835), Chinese (*n* = 415), Indian (*n* = 1772), Irish (*n* = 3779), and Pakistani (*n* = 654) [30].

### Indigenous community consultations

This project has been carried out in consultation and ongoing engagement with Tiwi Elders and lead Indigenous research experts. The present study adheres to all guidelines, such as those of the National Health and Medical Research Council (NHMRC), developed to steer the ethical conduct of research with Indigenous Australian people. The core values of Spirit and Integrity, Cultural Continuity, Equity, Reciprocity, Respect, and Responsibility have been embedded throughout the project*.* Participants provided consent for genetic samples to be used to investigate the causes of CKD at the time of collection as previously reported [25]. The current study subsequently received the support of the Tiwi Island Land Council.

### Analysis of phenotypic data

**Fig. 1:**
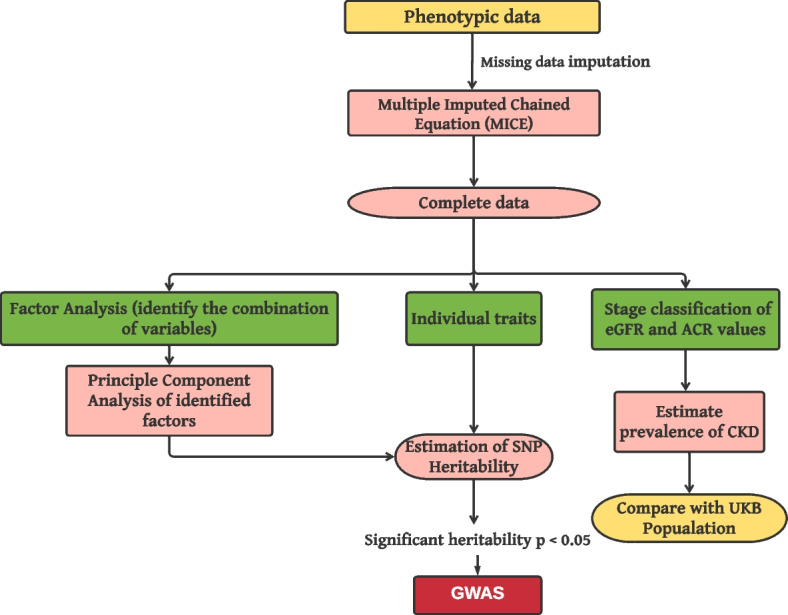
Methodology pipeline for data analysis. It involves missing data imputation, estimating the disease prevalence using ACR and eGFR, the relationship between the phenotypes, heritability estimation, and GWAS analysis. The phenotype data analysis was performed using R software v4.0.3, and heritability and GWAS analysis was performed using GCTA v1.93.2 functions *–reml* and *–mlma-loco* respectively

Few missing values were observed in the phenotype data, which were assumed to be missing at random. To obtain a complete dataset, we utilized the multivariate imputed by chained equation (MICE) technique [31] and employed R package mice [32]. The complete set of descriptive statistics associated with these data is given in Additional file 1: Table S1. We used Kidney Disease Improving Global Outcomes (KDIGO) nomenclature [8] to capture the prognosis of CKD using eGFR and ACR. CKD is classified into six categories based on eGFR, ranging from normal kidney function to kidney failure, as follows: G1 (≥ 90; Normal kidney function), G2 (60–89; mild loss), G3a (45–59; mild to moderate loss), G3b (30–44; moderate to severe loss), G4 (15–29; severe loss), and G5 (≤ 15; kidney failure). eGFR levels ≤ 60 or stage ≥ stage G3 indicate chronic kidney disease. The ACR stratification was as follows, stage A1 (< 3 mg/mmol; normal to mildly increased risk), stage A2 (3 to 30 mg/mmol; moderately increased risk), and stage A3 (> 30 mg/mmol; severely increased risk). To demonstrate the extent of kidney disease within the Tiwi population, we conducted a comparison of ACR and eGFR with the UKBB population. To ensure comparability between the two populations, we narrowed the Tiwi population study cohort to individuals between the ages of 37 and 73 for this comparison. This comparative cohort includes 279 individuals from the Tiwi and all UKBB cohorts divided into different ethnicities as mentioned in the Study populations and datasets section. The Kruskal–Wallis test was utilized to examine the difference of ACR and eGFR between the Tiwi population and various populations in UKBB data. It is important to note that this comparison cohort of the Tiwi was exclusively utilized to compare phenotypes. Additionally, we utilized ANCOVA (Analysis of Covariance) to compare the ACR among various ethnic groups, adjusting for covariates such as HbA1c and age. Subsequently, post-hoc analysis was carried out using the Bonferroni correction. The ANCOVA is a statistical method used for comparing the means of two or more groups while accounting for continuous covariates. The ANCOVA and post hoc test were carried out using the *Anova* function in the R car package [33] and *emmeans_test* from the R rstatix package [34] respectively. We then applied the Mann–Whitney *U* test to assess the statistically significant differences in ACR, eGFR, and A1c phenotypes between the diabetes and non-diabetes groups. We subsequently performed an exploratory factor analysis (EFA) to determine the relationship between the phenotypes, which helped us to identify the underlying hidden structure of a set of variables in the data [35]. And scree plot was used to decide the optimal number of factors required for further analysis. When the factor loadings of any variable exceeded 0.30, we considered the variable to be loaded onto that factor. If a variable was loaded onto multiple factors and its loadings exceeded 0.30, our determination of the factors was based on their correlation with other variables that were already loaded into the same factor. If the correlation was greater than ± 0.5, the variable remained within the factor; otherwise, the variable was allocated to another factor with the next highest loadings or a higher correlation with other variables within that factor. The identified factors were further used to define the variable groups for the multi-phenotype to-genotype association. A principal component analysis (PCA) enabled us to identify the key features that account for a significant portion of the variability in the data and are used to capture the pleiotropic loci in association with clinical data. PCA was performed on the set of phenotypes uncovered in the factor analysis, and only principal components (PCs) with an eigenvalue > 1 were used for association analysis. These computed PCs were used as an output variable within the frame of the GWAS analysis. Factor analysis and PCA were performed using the R package *FactomineR* [36]. The function *factanal* was used to perform factor analysis with *varimax* rotation and *five* factors, while the function *PCA* adjusted for age and sex was used for PCA analysis. Next, we estimated the SNP heritability $({h}_{SNP}^{2})$ for all individual traits and calculated PCs using Genome-wide Complex Trait Analysis (GCTA v1.93.2) *–reml* function [37]. Before estimating heritability, we log-transformed the skewed variables to reduce the skewness present in the phenotypes. GWAS was performed only on the traits with significant heritability (*p* < 0.05). The entire pipeline of phenotype data analysis is shown in Fig. 1.

### Genome-wide association analysis

The mixed-level linear model was performed for the traits (individual and phenotype PCs) to account for the cryptic relatedness (genetic relationship matrix (GRM)) between the individuals and other fixed covariates such as age, and gender to determine the association between the genotype and phenotype. To account for population stratification, genotype PCs were performed using PLINK v1.9 [28, 38], and the top two PCs were added as a covariate in the linear mixed model. We performed this model using the *–mlma-loco* function of GCTA v1.93.2. This GCTA-LOCO approach provides a more robust estimate by excluding the tested SNPs from calculating the GRM to avoid the loss of power and also helps to reduce the risk of false positives and false negatives in the association analysis [39]. We employed the mixed-level linear model to account for the substantial degree of relatedness among Tiwi individuals. For instance, the identity by descent (IBD) estimates showed that 45.2% of the individuals shared 1st-degree relations, while only 21.8% of the study cohort consisted of unrelated individuals. Genetic loci that passed the genome-wide significance *p*-value threshold of 5 × 10^−8^ with a cluster of at least two nearby linkage disequilibrium (LD) variants with *p* < 1 × 10^−5^ was considered statistically significant and investigated further in this study. The Manhattan plot was used to visualize the distribution of association of the genetic variant across the genomes. To assess the significance of genetic association in GWAS results, we utilized a QQ plot to compare the observed distribution of *p*-values to the expected distribution under a null model of no association. A deviation from the diagonal would indicate the presence of systematic error or bias in the association test. Genotype PC was involved in the model to account for the potential population stratification and correction factor lambda (λ) was estimated to check the presence of other sources of systematic error (λ = 1 indicates there is no population stratification and systematic error, λ < 1 or λ > 1 indicates that there is presence of population stratification and sources of systematic error). Additionally, we performed a conditional and joint association (cojo) analysis to capture the independent signal associated with each phenotype using the *–cojo-slct* from the GCTA v1.93.2 tool, uses stepwise model regression to mitigate high SNP dependency and identify LD-independent SNPs with an LD *r*^2^ < 0.1 [40].

We used ANNOVAR (version 2021Jul28) [41] to functionally annotate the identified loci. ANNOVAR is widely used to functionally annotate SNPs, indels (insertions and deletions), and copy number variation (CNVs) using up-to-date information from a wide variety of genomic databases and algorithms. We used the GWAS Catalog [42], Open Target Genetics [43], Type 2 Diabetes Knowledge Portal [44], Human Protein Atlas [45], and Harmonizome [46] to elucidate the roles of genes or variants associated with phenotypes in the present study. In addition, we assessed the extent of linkage disequilibrium (LD) for independent significant SNPs and obtained the high LD (> 0.25) variants. We then compared the above variants against the kidney eQTL results from Liu et al., [47] which encompassed eQTL outcomes for human kidneys (*N* = 686) and over a million significant SNP-gene pairs (FDR < 0.01) identified through a meta-analysis of four eQTL studies. Lastly, we compared the distribution of allele frequency to the control (i.e., UKBB) cohort to determine the extent of identified variants in other ethnic populations.

## Results

### ACR indicates that a significant proportion of the population is prone to kidney disease and has high heritability.

We used the key markers of kidney function eGFR and ACR to stratify CKD prognosis and assess the risk of kidney disease. Based on the eGFR (< 60 ml/min/1.73 m^2^) threshold for chronic kidney disease, our findings indicate that 5.7% (95% CI [3.8%, 8.1%], *p* < 0.001) of the study population exhibited signs of renal disease. In contrast, individuals in the Tiwi cohort with an ACR > 30 mg/mmol account for 20.3% of the population and are at high risk of CKD (Additional file 1: Table S2). Based on these two markers, we found that 20.9% (95% CI [17.4%, 24.8%], *p* < 0.001) of the Tiwi cohort was at high risk for developing CKD (Additional file 1: Table S2). From this sub-cohort of 21%, 44% exhibited HbA1c (hemoglobin A1c) levels above the diabetes-definition threshold of 6.5% and 35% had pre-diabetic definition levels (5.7% to 6.4%).

**Fig. 2:**
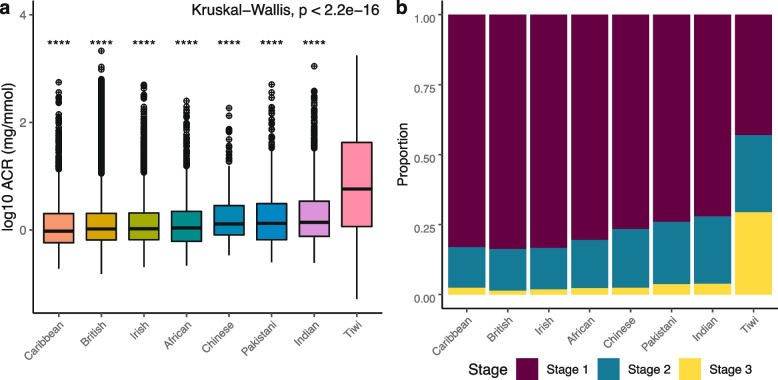
**a** Comparison of ACR between populations—Represent the comparison of urinary Albumin-to-creatinine ratio (ACR) between the Tiwi and control UK Biobank populations and the Kruskal–Wallis test was performed to compare the median ACR between the population. Log transformation was applied to ACR values for visualization. And stars on the plot represent the significance level (**** −  < 0.0001). **b** Proportion of ACR between population—Compare the proportion of ACR stages between the population. Individuals in stage 1 have less to moderate risk, stage 2 has moderate to severe risk, and stage 3 has a severely increased risk of developing CKD

We next compared ACR and eGFR between the Tiwi cohort with the UKBB population. To ensure comparability between the two populations, we narrowed down the Tiwi population study cohort to individuals aged between 37 and 73. We observed significantly higher ACR values in the Tiwi cohort (*p* < 2.2e − 16), indicating an increased risk of developing end-stage kidney disease compared to the UKBB population (Fig. 2). In particular, the median (Q1, Q3) for ACR in the Tiwi cohort was 5.77 (1.2, 42.5) which was significantly higher than the British (1.1 (0.7, 2.1)). A stage classification of ACR in the Tiwi and UKBB populations revealed that a remarkably higher proportion of the Tiwi (29.4%) are at severely increased risk of developing CKD compared to other ethnic populations (British 1.35%, Caribbean 2.45%, African 2.27%, Irish 1.88%, Chinese 2.40%, Indian 3.83%, and Pakistani 3.7%) (Fig. 2b). Upon further investigation, the ANCOVA analysis revealed a significant difference in ACR among ethnic groups while adjusting for HbA1c levels. And the F-ratio was 273.2 with the significance value was less than 0.001. The post-hoc test further indicated a significant difference between the Tiwi and other ethnic groups (*p* < 0.001). In contrast, we observed no significant differences in eGFR between the Tiwi and UKBB cohorts. These findings indicate that ACR is a potential early biomarker for identifying individuals at high risk of renal disease in the Tiwi community.

SNP-based heritability was estimated for both ACR and eGFR. The logarithmic value of ACR was found to have a heritability of 52.6% (95% CI [34.3, 70.9%], *p* < 0.001) indicating ~ 1/2 of the variance of ACR in this population is influenced by genetic factors, and was the highest of all kidney-related phenotypes (Additional file 1: Table S3). In contrast, the least heritability was observed for eGFR (20.5%; 95% CI [3.7–36.8%], *p* < 0.001). This heritability warrants GWASs of these phenotypes to search for genetic markers of kidney disease.

### Identification of multiple phenotype factors contributing to renal dysfunction

Using an exploratory factor analysis, we retained five factors based on the scree plot and utilized them for further analysis. Factor 1 was loaded with weight, waist, body mass index, HbA1c, and serum albumin, and accounted for 18% of the total variance. Other than serum albumin, Factor 1 did not exhibit a direct relationship to kidney disease. On the other hand, Factor 2 encompassed well-established kidney markers, such as eGFR, ACR, serum creatinine, and urine albumin, with respective factor loadings of − 0.58, 0.87, 0.73, and 0.88. Factor 2 accounted for approximately 17% of the total variance (Additional file 1: Table S4 and Figure S1). Notably, Factor 2 displayed a positive association with all phenotypes, except for eGFR. The elevated levels of ACR, urine albumin, serum creatinine, and reduced eGFR levels are indicators of renal dysfunction. Our GWAS analysis focused primarily on the combination of these four renal phenotypes. Furthermore, urine osmolality (factor loading = 0.76) and urine creatinine (factor loading = 0.97) were strongly associated with Factor 3, which was also associated with kidney function. However, unlike Factor 2, Factor 3 did not exhibit diagnostic value on its own. For instance, urine creatinine and urine osmolality are used to standardize the assay results for urinary dilution and do not serve as indicators of kidney disease. Blood pressure variables including systolic and diastolic blood pressure were strongly associated with Factor 4, with factor loadings of 0.79 and 0.78, respectively. Uric acid and height were associated with Factor 5, with corresponding loadings of 0.37 and 0.99 respectively. Factor score loadings and the grouping of the phenotypes are given in the Additional file 1: Table S4.

Following the identification of Factor 2 via EFA, we performed a PCA for kidney function traits (i.e., Factor 2). These primary traits (i.e., ACR, eGFR, urine albumin, and serum creatinine) of Factor 2 produced 4 PCs in total. The first PC (CGAA_PC_1), with an eigenvalue > 1, accounted for 70.9% of the total variance present in the phenotype data. The remaining PCs (i.e., CGAA_PC_2, CGAA_PC_3, and CGAA_PC_4) exhibited eigenvalues < 1 and were not considered for further analysis. In line with the factor analysis, PC1 exhibited significantly positive associations with ACR (*r* = 0.86; *p* < 0.001), serum creatinine (*r* = 0.86; *p* < 0.001), and urine albumin (*r* = 0.88, *e* < 0.001), and a significantly negative correlation with eGFR (*r* =  − 0.77; *p* < 0.001) (Table 1). Low eGFR and high levels of ACR, creatinine, and albumin are indicators of reduced kidney function and potential renal dysfunction. Heritability for CGAA_PC_1 was found to be 0.21 (*p* < 0.001) after adjusting for age and gender*.* GWAS was subsequently performed for CGAA_PC_1 to elucidate the collective impact of these primary traits on genotype data.

**Table 1:** Loading score, significant correlation coefficient with phenotype, heritability estimates, and corresponding measures of significance

PC	Eigen value	% var	Correlation coefficient^*^				Heritability	
			**Urine albumin**	**Serum creatinine**	**ACR**	**eGFR**	**h**^**2**^** (SE)**	***p*-value**
CGAA_PC_1	2.84	70.95	0.88	0.86	0.86	− 0.77	0.21 (0.083)	3.41 × 10^−4#^
CGAA_PC_2	0.65	16.10	0.32	− 0.23	0.41	0.57	0.15 (0.086)	1.28 × 10^−2^
CGAA_PC_3	0.32	8.02	− 0.19	0.45	-	0.29	0.00 (0.07)	> 0.05
CGAA_PC_4	0.19	4.92	0.30	-	− 0.31	-	0.17 (0.082)	6.39 × 10^−3#^

^*^-reported significant correlation only

^#^**-**statistically significant

*SE* standard error, *% var* percentage of variance explained

### ACR identifies population-specific alleles and exhibits different genomic architecture

**Fig. 3:**
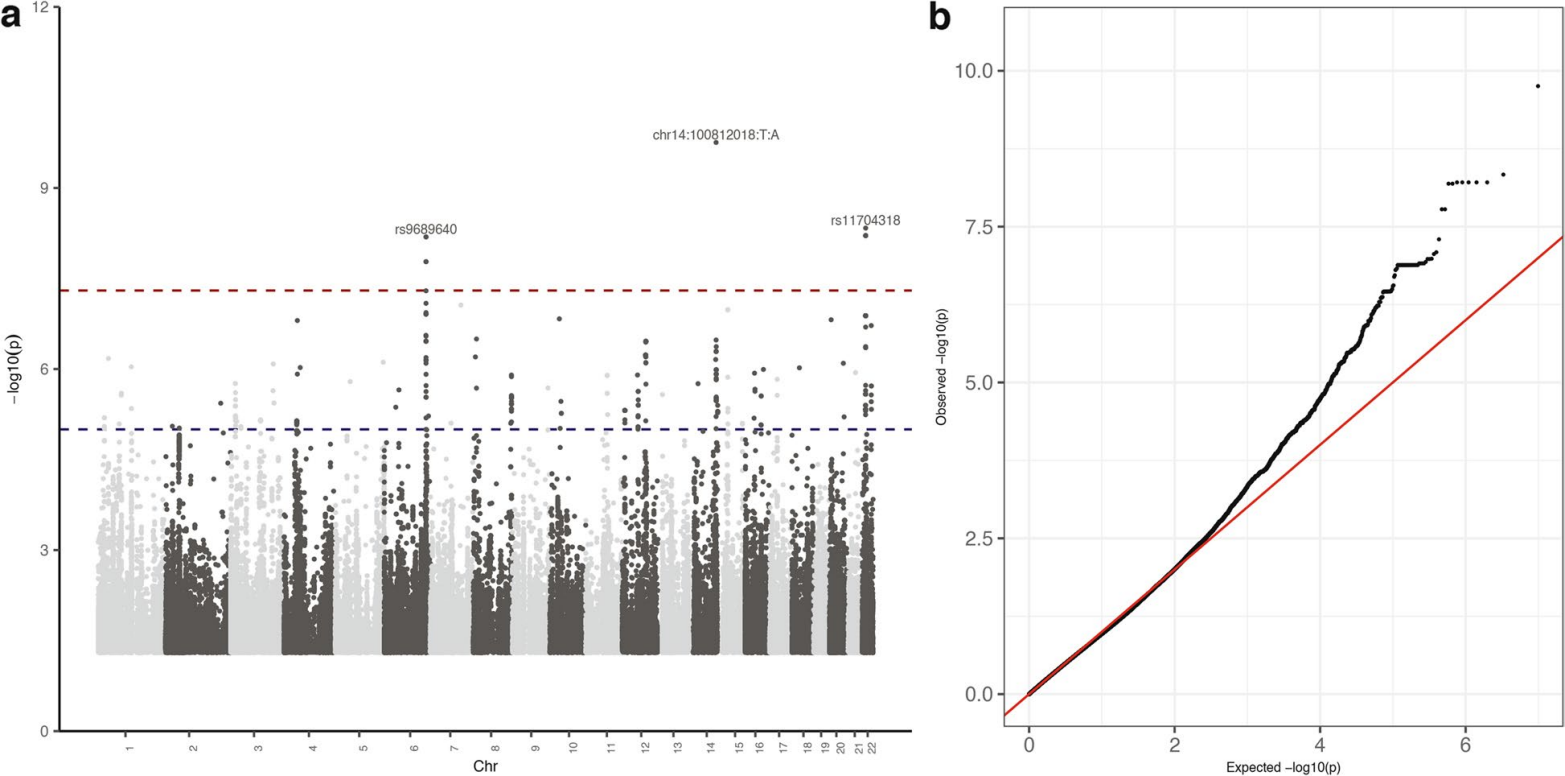
**a** Manhattan plot for the phenotype albumin-to-creatinine ratio (ACR). The red dashed line indicates the genome-wide significance threshold (*p* < 5e − 8), while the blue dashed line indicates the nominal significance level (*p* < 1e − 5). **b**) The QQ plot for the trait ACR. The genomic inflation factor (λ) is equal to 1.004 (i.e., mostly equal to 1) and indicates that the significant variant is not due to chance and thus could be considered a causal variant for the kidney functional trait

As ACR was determined to be the most heritable component, a GWAS was performed for ACR to identify associated variants in individuals at high risk for kidney disease in the population. We used a mixed-linear model to adjust for age, sex, and population structure (i.e., genotypes PC1 and PC2) as fixed factors, with genomic relatedness and other SNPs as random factors. The significance levels of the SNPs associated with ACR throughout the genome are shown in Fig. 3a. The GWAS analysis of ACR revealed a genomic inflation (λ) of 1.004, suggesting no significant inflation at the association level. This indicates that the observed significant associations are unlikely to be attributed to chance, population stratification, or systematic biases (Fig. 3b).

The SNP that is most significantly associated with ACR—*14:100812018* (AF = 5.14%; *b* = 0.857; *p* = 1.76 × 10^−10^) is in region 14q32.2 and mapped to the oncogenic long non-coding RNA (lncRNA) gene *MEG3* (maternally expressed imprinted gene 3). Upon further investigation, we found that this novel variant was absent in all UKBB cohorts (i.e., British, Caribbean, African, Chinese, Indian, Irish, and Pakistani). Additionally, we identified another SNP—rs9689640 (AF = 94.70%; *b* =  − 0.874; *p* = 6.47 × 10^−9^) in genomic region 6q25.2, which mapped to an intronic region of the T-cell lymphoma invasion and metastasis 2 gene (*TIAM2*)*.* The allelic frequency of this variant was remarkably high in the Tiwi population (AF = 94.70%). Similar frequencies were observed in the African (96.2%) and Caribbean (93.3%) populations, whereas the frequency in other populations was substantially lower (i.e., British: 77.42%, Chinese: 65.03%, Indian: 67.33%, and Irish: 76.04%) (Additional file 1: Table S5).

**Fig. 4:**
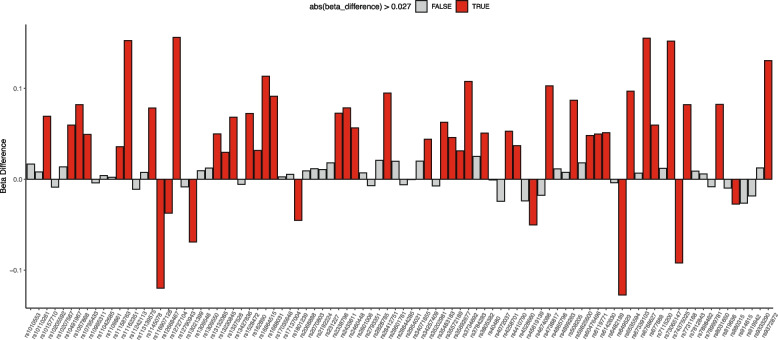
Beta difference of known genome-wide significant variant associated with ACR between the Tiwi and published GWAS results from the GWAS Catalog. The beta difference is the absolute difference between the Tiwi and published results. The red-colored bar indicates the beta difference is greater than the mean beta difference (0.027), while the gray color represents the beta difference is less than the mean beta difference

In addition, we observed another significant SNP—rs11704318 (AF = 6.40%; b = 0.792; *p* = 4.26 × 10^−9^) in the genomic region 22q11.23 which belongs to 3`UTR of *RAB36* gene. The gene *RAB36* a member of the RAS oncogene family is believed to be involved in protein transport and enables the guanisine-5′-triphosphate (GTP) binding activity and GTPase activity. This variant was found to be significantly more abundant in the Tiwi (6.40%) in comparison to the UKBB population (i.e., British 0.20%, Caribbean 0.04%, African 0%, Chinese 0.78%, Indian 0.32%, Irish 0.28%, and Pakistani 0.42%) (Additional file 1: Table S5). The independent SNPs that surpass the genome-wide significance level (*p* < 5 × 10^−8^) are given in Table 2, while SNPs that pass the nominal significance level (*p* < 1e − 5) can be found in the Additional file 2. We subsequently conducted a comparison of the effect size (beta coefficients) of established genome-wide significant variants associated with ACR in the Tiwi population with other GWAS findings listed in the GWAS Catalog [42]. As a result, we observed that the average absolute difference in beta coefficients between the Tiwi population and other populations was 0.027, with a standard deviation of 0.053 (Fig. 4). For further examination, we verified the co-localization of our independent SNPs with high LD (> 0.25) SNPs in the kidney eQTL result reported by Liu et al. [47]. Our investigation did not reveal any corresponding matches, suggesting a lack of association between identified genetic variants and gene expression in the kidney. For instance, the first independent variant (chr14:100812018:T:A) in the *MEG3* gene, exhibited a high LD with 127 nearby variants, none of which were found in the kidney eQTL results. This pattern also held for the remaining identified variant as well.

**Table 2:** Genome-wide significant independent loci (*p* < 5e − 8) associated with the albumin-to-creatinine ratio (ACR) phenotype

*ID*^*a*^	*Chr*	*Position*	*Alt allele*	*Allele frequency*	*Effect size*	*Std. error*	*p*	*Nearest gene*
Chr14:100812018:T:A	14	100812018	A	0.051	0.857	0.134	1.76 × 10^−10^	MEG3
rs11704318	22	23164355	G	0.064	0.792	0.135	4.62 × 10^−9^	RAB36
rs9689640	6	154966925	C	0.947	− 0.874	0.151	6.47 × 10^−9^	TIAM2

^a^Variants do not have rsIDs are given a ID in the format C*hr:Pos:Ref:Alt*

### Multiple phenotype association reveals the presence of pleiotropic loci

**Fig. 5:**
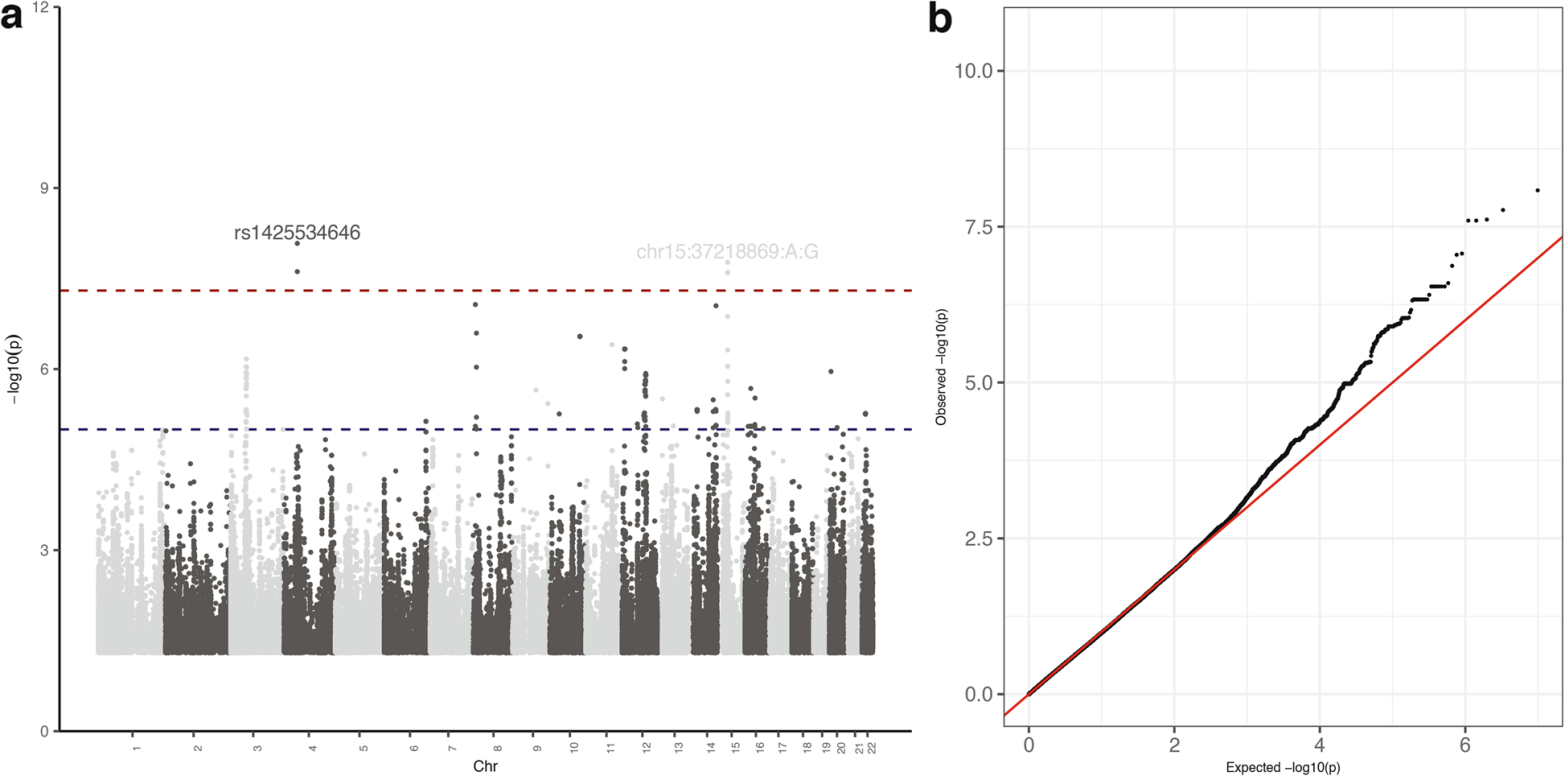
**a** Manhattan plot for the first PCA components in multiple phenotype analysis, i.e., CGAA PC 1. The Red dashed line indicates the genome-wide significance (*p* < 5e − 8), while the blue dashed line indicates the nominal significance level (*p* < 1e − 5). **b** The QQ plot for the trait CGAA PC 1. The genomic inflation factor (λ) is equal to 1.01 (mostly equal to 1) which shows that the significant variant is not due to chance, it might act as a causal variant for the given traits

**Fig. 6:**
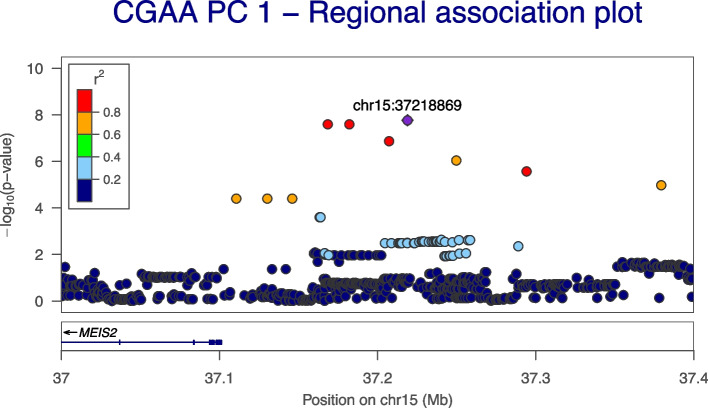
Regional association plot for the associated region on chromosome 15q14. The significance level is given on the *y*-axis and the genomic region is on the *x*-axis. The lead SNP was indicated using the violet color and pairwise LD between the tagging SNP and other SNP is indicated by color

We carried out a GWAS on the traits (i.e., PCs) identified in the present study to capture the pleiotropic effect locus, which is the combined impact of multiple phenotypes (i.e., ACR, eGFR, urine albumin, and serum creatinine) on genotype. The GWAS accounted for sex, age, population stratification, and cryptic relatedness. The first PC (CGAA_PC_1) exhibited association peaks that exceeded the genome-wide statistical significance threshold (*p* < 5 × 10^−8^). The two most significant peaks are located on chromosome 15q14 and at the end of chromosome 4p12 (Table 3 and Fig. 5a). The QQ plot (Fig. 5b) for this trait further demonstrates that the significant variants observed might be owing to the trait effect and not due to chance. Figure 6 shows the regional association plot for the region of interest on chromosome 15, and indicates that there is a cluster of SNPs with high LD that pass either genome-wide or nominal significance levels, thereby leading to the discovery of independent SNP Ch*r15:37218869:A:G* (AF = 6.15%; beta = 1.25; *p* = 1.71 × 10^−8^). This novel variant is in an intergenic region approximately 117 kb upstream of the protein-coding gene *MEIS2* (Meis Homeobox 2). The next closest protein-coding gene to the SNP is more than 500 kb away downstream of *MEIS2*. Furthermore, this variant is completely absent in the UKBB populations (Additional file 1: Table S5).

**Table 3:** Genome-wide independent loci (*p* < 5e − 8) that are significantly associated with the multi-phenotype traits (i.e., CGAA_PC_1: ACR, eGFR, urine albumin, and serum creatinine)

*ID*^*a*^	*Chr*	*Position*	*Alt allele*	*Allele frequency*	*Effect size*	*Std. error*	*p*	*Nearest gene*
rs1425534646	4	49304522	A	0.051	1.257	0.218	8.27 × 10^–9^	CWH43 (242 kb)
Chr15:37218869:A:G	15	37218869	G	0.062	1.252	0.222	1.71 × 10^–8^	MEIS2 (117 kb)

^a^Variants do not have rsIDs are given a ID in the format C*hr:Pos:Ref:Alt*

Lastly, the GWAS of the CGAA_PC_1 also identified the SNP rs1425534646, which lies 242 kb downstream from the protein-coding gene *CWH43* located in the genomic region of 4p12. This gene is predicted to be involved in glycosylphosphatidylinositol anchor biosynthetic processes, which are lipid anchors for many cell surface proteins and are integral components of cell membranes. A small number of variants (rs11725397; beta = 0.0018, *p* = 4 × 10^−12^) in this gene are related to eGFR in the European population and this variant is approximately 287 kb away from the lead SNPs [15]. The SNP that surpasses the nominal significant level is given in Additional file 3.

### Association between CKD and diabetes

The prevalence of diabetes is estimated to be 24.39% (95% CI [20.57, 28.66]) in the Tiwi population. A significant difference in the glycaemic indicator HbA1c was observed between the diabetic and non-diabetic cohorts (*p* < 0.05). However, it is worth noting that the average HbA1c value of the non-diabetic cohort falls within the pre-diabetic range (Table 4). In addition, we correlated HbA1c levels with kidney biomarkers. HbA1c exhibited a positive correlation with ACR (*r* = 0.44, *p* < 0.05) indicating a correspondence of high blood glucose and albuminuria. Conversely, HbA1c displayed a negative correlation with eGFR, exhibiting the inverse relationship between glycaemic control and renal function (*r* =  − 0.31, *p* < 0.05). To provide further insight into these differential relationships, we examined the differences in ACR and eGFR among diabetes and non-diabetes groups, as outlined in Table 4. Notably, the median eGFR levels in both diabetic and non-diabetic groups exceeded 90, indicating normal kidney function. However, more concerning is ACR levels were more concerning, and there was a huge disparity in the diabetic group. Individuals with diabetes exhibited significantly higher ACR values, which indicates the presence of albuminuria and early signs of kidney damage. In addition, we conducted a GWAS analysis for HbA1c (h^2^ = 0.117, 95% CI [0.00, 2.72], *p* = 0.044) and diabetes status. Unlike the ACR, no significant hits were identified for either of these diabetic phenotypes. Furthermore, to establish a connection between diabetes and renal disease, we investigated the association between the identified risk SNPs and HbA1c GWAS results. However, no statistically significant association was found. For instance, the risk variant *chr15:37218869:G:A* identified in multiple phenotype analysis (CGAA_PC_1) located near the *MEIS2* gene (*b* = 0.24, std.error = 0.13, *p* = 0.061) tends to increase HbA1c levels but did not reach either genome-wide or nominal significance (Additional file 1: Table S6).

**Table 4:** Comparison of established kidney markers (ACR and eGFR) and HbA1c among the diabetes/non-diabetes group. A Mann–Whitney *U* test was used to assess statistical significance between the groups

Traits	Diabetes—median (Q1, Q3)		*p*-value
	**Yes (*n* = 111)**	**No (*n* = 344)**	
ACR (mg/mmol)	30.88 (5.19, 95.34)	1.25 (0.52, 6.59)	< 0.001^*^
eGFR (ml/min/1.73 m^2^)	102.26 (77.12, 113.19)	109.84 (96.84, 120.49)	< 0.001^*^
HbA1c (%)	7.30 (6.60, 10.05)	5.70 (5.50, 5.90)	< 0.001^*^

^*^statistically significant; Q1: 1st quartile; Q3: 3rd quartile

## Discussion

The present study is the largest genetic study in this underrepresented Indigenous population to date. We combined collected clinical data with WGS techniques to elucidate the underlying genetic mechanisms of CKD in this underrepresented population. As a result, we found that a high proportion of the Tiwi population is at high risk for CKD using ACR compared to the UKBB population and that ACR levels could serve as a biomarker to identify high-risk individuals. Furthermore, the noted disparity in ACR between the Tiwi and other ethnic groups remained independent of HbA1c levels and thus glycemic control. We also show that Tiwi may be genetically predisposed to high ACR levels and found several genetic variants associated with kidney function that are novel to the Tiwi population. Our findings also indicate that the genetic architecture for ACR in this population is notably different from that observed in other populations. Furthermore, our examination of multiple traits revealed a novel pleiotropic locus in the *MEIS2* gene. In the present study, we aimed to identify both clinical and genetic factors associated with renal function/dysfunction using approximately 20% of the total Tiwi population. Despite the prevalence of CKD in the Tiwi people, there has been limited research on the genetics of kidney disease in this population. Previous studies have indicated that renal disease is highly heritable in Aboriginal Australians, suggesting that some individuals may be inherently susceptible to the disease [25, 27]. Here, we found that ACR has a high degree of heritability (52%), while a previous study on Indigenous Australians found that ACR explained 64% of heritability [27]. Additionally, as we found that ACR was positively correlated with urine albumin and serum creatinine, and negatively correlated with eGFR, we performed a GWAS for ACR and a collective impact of kidney phenotype that included ACR, eGFR, urine albumin, and serum creatinine (multi-phenotype). As a result, we identified three SNPs that were independently associated with the single measure ACR and two SNPs that were independently associated with the collective impact of multiple phenotypes. Furthermore, we identified a population-specific variant associated with renal function, which significantly differs in allele frequency compared to UKBB populations.

As we found that 5.7% of the Tiwi individuals in the present study had eGFR < 60 mL/min/1.73 m^2^, we believe that using the standard eGFR threshold for kidney disease (i.e., eGFR < 60 mL/min/1.73 m^2^) underestimates the prevalence of renal disease in the Tiwi community. This is in concordance with previous findings [12, 25, 48], in which individuals exhibit a severe kidney impairment before experiencing a decline in eGFR levels, resulting in the late-stage manifestation of advanced renal disease progression in the Tiwi population [49]. Additionally, not only did the Tiwi population in the present study exhibit significantly higher ACR than the UKBB population independent of HbA1c levels, ACR also exhibited a remarkably higher heritability in comparison to eGFR and other phenotypes. The disparity between the high degree of albuminuria and the lower prevalence of CKD estimated by eGFR may be attributed to the higher occurrence of early-stage kidney disease, where kidneys maintain filtration but become more permeable to albumin, leading to albuminuria [49]. This likely reflects the hyperperfusion of still functioning glomeruli in the presence of an ongoing paucity or loss of other glomeruli. As the compensatory hyperperfusion reaches its limits, eGFR experiences a sharp decline, resulting in a late manifestation of the CKD process. Thus, ACR could be a more reliable marker for identifying high-risk individuals in the earlier stages of kidney disease, in agreement with previous findings in Indigenous Australians [25, 49, 50]. As diabetes is a risk factor for CKD, there is an inverse relationship between the HbA1c and eGFR and a positive relationship between ACR and HbA1c. The individuals with higher HbA1c, have an elevated ACR value and declined eGFR values, reflecting reduced kidney function. Furthermore, notable distinctions in ACR and eGFR were observed between diabetic and non-diabetic cohorts. Nevertheless, the comparison of eGFR between the diabetes and non-diabetes cohorts revealed no clinical significance. In both cohorts, eGFR values surpassed 90, indicative of normal kidney functions. These relationships underscore the importance of glycaemic control in preserving kidney health in these populations.

To elucidate the relationship between the genotype and phenotype in CKD, we carried out the GWAS study for the phenotype ACR and a multi-phenotypic GWAS that included ACR, eGFR, urine albumin, and serum creatinine. The GWAS for the phenotype ACR identified a highly significant novel SNP in the lncRNA tumor suppressor gene *MEG3*, which was absent in all UKBB cohorts. The gene *MEG3* is known to be a significant mediator of ischemia–reperfusion injury (IRI)-induced acute kidney injury (AKI), where it is upregulated in the renal cortex in IRI mice and exacerbates IR-induced AKI [51]. Furthermore, suppressing *MEG3* expression inhibited the apoptosis of proximal tubular cells, rescued elevated levels of serum blood urea nitrogen (BUN) and creatinine, and decreased the number of damaged tubules following IRI [51]. The *MEG3* gene also controls the pyroptosis of tubular epithelial cells, which is critical in sepsis-related acute kidney damage [52] induces the accumulation of p53 protein and selectively regulates the expression of p53 target genes [53]. It is also noteworthy that p53 polymorphisms are directly associated with ACR (*p* = 0.01) in Indigenous Australians living in the East Arnhem region of Australia [27, 50]. Nevertheless, these populations share a geographic similarity with the Tiwi individuals as they all are from the Northern Territory, Australia. This study also found that the p53 genotype is also associated with increasing HbA1c (*p* = 0.01) but independent of ACR. These findings suggest that *MEG3* may play a role in the pathogenesis of kidney diseases such as AKI and CKD. Owing to the complexity of kidney disease, it is tempting to speculate the detailed function of renal disease-associated variants present in this MEG3 gene; however, further research will be necessary to elucidate the role of *MEG3* in CKD in the Tiwi population.

The next independent SNP associated with ACR lies in the intronic region of the *TIAM2* gene in chromosome 6q25.2. *TIAM2* encodes a guanine nucleotide exchange factor that plays a role in activating RHO-GTPases, is an upstream regulator in the Rac pathway, and is involved in cellular proliferation, cellular migration, and invasion in multiple types of cancer [54, 55]. This gene is also is expressed in kidney tissue, particularly in proximal tubular and B-cells, and believed to play an important role in neural cell development [45]. This variant is significantly more prevalent in the Tiwi population compared to British, Irish, and Indian subpopulations of the UKBB cohort, although it was similar in African and Caribbean subpopulations. This SNP (rs9689640) was found to exhibit glomerular-specific differential expression [56] and was associated with end-stage renal disease (OR = 0.9085, *p* = 0.024) in large-scale GWAS studies of mixed ancestry, as well as in comparison of end-stage renal disease vs macroalbuminuria (OR = 0.883, *p* = 0.022) [57, 58]. The beta coefficients from the Sandholm et al. study results were consistent with our findings and serve as a protective factor. In addition, a GWAS research including 1700 people of European origin discovered that this polymorphism was linked to diabetic retinopathy (OR = 1.296, *p* = 2.9 × 10^−5^) [59]. Furthermore, according to the Harmonizome database, there appears to be a functional connection between TIAM2 and kidney disease [46]. This association has been assigned a relative strength of approximately 1.104, which was calculated using standardized empirical *p*-values derived from publicly available association databases [46]. Therefore, we hypothesized that the *TIAM2*-associated variant identified in this population potentially contributes to regulating kidney function or pathogenesis of kidney disease.

The GWAS of the phenotype ACR also implicated a locus in the 3ʹUTR region of *RAB36*, a member of the RAS oncogene family. This variant was significantly associated with coronary artery disease (OR = 0.8663, *p* = 0.0038) in a previous GWAS study of over a million individuals [60] and has also been shown to be correlated with kidney disease and cardiovascular disease (CVD) [61, 62]. These previous findings indicate that this variant could potentially have an indirect impact on the pathogenesis of kidney disease. Additionally, this gene is predicted to be involved in the metabolism of proteins and vesicle-mediated transport. *RAB36* has also been shown to be highly expressed in all of the tissues assayed, most notably in the testis and brain, and is observed to prognostic factor in multiple cancer lines, particularly in renal cancer [45]. Coronary heart disease is a well-known independent risk factor for the progression of CKD to end-stage renal disease. In terms of cardiovascular diseases and renal disease progression, patients with coronary artery disease and diabetic nephropathy fall into the extremely susceptible group [61]. The Australia Institute of Health and Welfare (AIHW) has reported that Indigenous adults experience higher rates of CVD compared to non-Indigenous adults, with rates of 27% and 21%, respectively. Furthermore, the disparity of CVD rates between the Indigenous and non-Indigenous populations tends to increase as individuals age [63]. Thus, we hypothesize that kidney disease in the Tiwi cohort may have been affected indirectly via altered *RAB36* expression or a *RAB36* gene product. Furthermore, we compared the obtained GWAS results for ACR with known variants reported in the GWAS Catalog, this analysis revealed that none of the known kidney-associated variants attained either genome-wide or nominal significance levels. Additionally, we did not obtain any corresponding matches against the eQTL comparison, indicating a unique genetic architecture for kidney disease in the Tiwi population that differs significantly from other populations or is possibly attributed to the limited sample size and statistical power.

The multi-phenotype GWAS analysis (i.e., CGAA_PC_1) computed using phenotypes including eGFR, ACR, serum creatinine, and urine albumin implicated a novel variant located in an intergenic region approximately 117 kb upstream of the protein-coding gene *MEIS2*. Interestingly, while this variant lacked sufficient statistical power to attain the level of genome-wide significance in the individual phenotype GWAS analysis, it did achieve genome-wide significance when examined in the context of multiple phenotype GWAS analyses. *MEIS2* encodes a homeobox protein belonging to the three amino acid loop extension family of homeodomain-containing transcription factors and important regulators of cell proliferation during development. *MEIS2* is highly expressed in the glomeruli tissue of the kidney as compared to the tubulous [45] and has also been shown to play a significant role in the formation of new blood vessels [64]. This gene acts as a candidate marker gene for mesangial cell in mice, which is in the interpapillary space and regulate the glomerular filtration rate [65]. The transcription factor *MEIS2* also plays a role in disorders such as cardiac defects and intellectual disability [66, 67]. Furthermore, a GWAS demonstrated that *MEIS2* variants were associated with triglycerides using a mixed ancestry of millions of individuals [68, 69], as triglycerides are known to be one of the major risk factors for CKD. In particular, individuals with high triglycerides were 1.5 times more likely to experience loss in renal function, even after adjusting for factors such as sex, race, age, systolic blood pressure, diabetes status, and type of blood pressure medication taken [70]. *MEIS2* has also been shown to be associated with increasing let-7 family members in differentiating or aging nephron progenitor cells and is strongly upregulated in nephron progenitors and the renal stroma during kidney development [71]. Lastly, *MEIS1* is a paralog of *MEIS2* and is known to code for a protein analogous to *MEIS2* in both mice and humans [72]. *MEIS1* is highly expressed in the stroma and myofibroblasts of mouse and human kidneys and is upregulated in kidney myofibroblasts as a function of age and IRI, although it was not necessary for normal kidney function or the development of fibrosis [73]. *MEIS1*, along with *VEGFR-2*, was significantly downregulated in early-stage kidney cancer tissues compared to adjacent normal tissues [74]. Additionally, *MEIS1* was also downregulated in a variety of tumors, where downregulation was linked to the immune infiltration level of cancer patients and low expression predicted poor overall survival in kidney renal clear cell carcinoma and various other cancers [60]. Although there is no direct association between kidney function and the paralogs *MEIS1* and *MEIS2*, however, these genes appear to be expressed in kidney tissues and play a role in kidney development. Additionally, we examined the effect size and significance levels of identified risk SNPs linked to HbA1c levels, none of the risk variants reached either genome-wide or nominal significance. Despite the absence of statistical significance, it is noteworthy that the effect sizes of HbA1c, influenced by the risk SNPs, remained consistent with those observed for the SNPs identified via GWAS of multiple traits. These findings highlight the lack of relevance and influence of the SNPs on HbA1c levels. We note that the lack of significance may be attributed to the statistical power or limited sample size. Further functional studies will be necessary to determine their role in kidney function and its associated functions.

Limitations of our study include the relatively low sample size and it is possible that some SNPs did not reach genome-wide significance in the GWAS. Secondly, Validation for the GWAS can be achieved through replication analysis on a similar cohort. However, in this case, the distinct genomic characteristics of the Tiwi cohort make it challenging to conduct such a replication analysis. Finally, our GWAS analysis did not account for environmental factors. However, our study provides valuable insights into the genetic basis of the phenotype of interest. Future research that incorporates environmental data could further elucidate the interplay between genetics and environment in the development of this phenotype.

## Conclusions

In conclusion, our study demonstrates that ACR serves as a reliable predictive marker for CKD risk in the Tiwi population and that the Tiwi population carries a population-specific CKD allele, warranting further research to elucidate the functional significance of this variant. The discovery of Tiwi-specific novel associations for several genes using ACR as a marker and the pleiotropic effect in the region 15q14 (*MEIS2*) using multiple phenotypes indicate the need for further genetic studies in this population. The identification of novel variants offers a potential means of screening individuals in this population to identify those at risk of kidney disease. Our results emphasize the significance of investigating population-specific genetic variations in underrepresented communities, which could play a crucial role in understanding disease susceptibility and developing personalized medicine approaches. Combining genomic data with corresponding clinical data represents an invaluable resource that can be harnessed to improve health for all Indigenous Australian populations while providing a roadmap addressing inequities in care access for this underrepresented population.

### Supplementary Information

**Additional file 1: Table S1.** The summary statistics for all the available phenotypes (N = 492) including kidney function, blood pressure, BMI, diabetes, etc. **Table S2.** Risk of CKD in the Tiwi population – Stage classification of eGFR and ACR. a) Stage classification using eGFR. b) Stage classification of kidney function using ACR. c) Risk status stratification using the combination of eGFR and ACR using KDIGO 2020 nomenclature. **Table S3.** The table shows the narrow sense SNP based heritability for the kidney phenotype available in the phenotype data along with its 95% confidence interval and significance level. Significance level less than 0.05 consider to significant heritable trait. **Table S4.** Factor score loadings for the different phenotypes involved in the factor analysis. The grouped variables are considered for the multiple phenotype GWAS association analysis and are indicated using different colors. **Table S5.** Allele Frequency in the UKBB population for independent SNPs and Tiwi population. **Table S6.** Effect size of HbA1c GWAS with identified risk SNPs. **Figure S1.** Correlation heatmap plot for the available phenotypes in the clinical data. The color was given based on the absolute correlation value. The right-side stacked bar chart represents the factor loading score observed in EFA The x-axis of the plot represents the factor loading scores.

**Additional file 2: Table S5a.** This table contains the variants which pass the nominal significance levels (p<1e-05) for the variable urinary Albumin-to-creatine ratio (ACR) and its annotation which includes consequences of the variant, mapped gene, beta coefficients direction (incline/decline).

**Additional file 3:** **Table S5b.** This table contains the variants which pass the nominal significance levels (p<1e-05) for the variable CGAA_PC_1 (multiple phenotype PCA factor) and its annotation which includes consequences of the variant, mapped gene, beta coeffecients direction (incline/decline).

## Data Availability

The Tiwi data are stored in the QUT HPC Infrastructure in Brisbane. We have established a Data Access Advisory Committee (DAC), comprising Elders, Tiwi community members, and study investigators. Requests for external research access to Tiwi data will be evaluated by the DAC on a case-by-case basis, and access will be granted accordingly. The timeframe for assessing applications for data access is typically 3 to 4 months. This stringent review process is aligned with the strong recommendation from the Tiwi community and the Ethics Committee, which approved this research, to control and restrict access to Tiwi data. Requests to access the datasets should be directed to A/Prof Shiv Nagaraj (shiv.nagaraj@qut.edu.au).

## Abbreviations

CKD: Chronic kidney disease
BVD: Background variant databases
gnomAD: Genome aggregation database
NHMRC: National Health and Medical Research Council
ACR: Albumin-to-creatinine ratio
eGFR: Estimated glomerular filtration rate
GWAS: Genome-wide association studies
OR: Odds ratio
CKDGen: Chronic Kidney Disease Genetic Consortium
QC: Quality control
SNPs: Single nucleotide polymorphisms
AF: Allele frequency
UKBB: UK Biobank database
PCA: Principal component analysis
EFA: Exploratory factor analysis
MICE: Multiple imputed chained equation
GCTA: Genome-wide Complex Trait Analysis
3′UTR: Three prime untranslated region
GRM: Genetic relationship matrix
GTP: Guanisine-5′-triphosphate
