# Supplementary material for: Novel genetic markers for chronic kidney disease in a geographically isolated population of Indigenous Australians: Individual and multiple phenotype genome-wide association study
Source: Genome Med. 2024 Feb 12;16:29. doi: 10.1186/s13073-024-01299-3 (PMC10860247; doi:10.1186/s13073-024-01299-3)
Supplement: Supplementary file 1 — Additional file 1: Table S1. The summary statistics for all the available phenotypes (N = 492) including kidney function, blood pressure, BMI, diabetes, etc. Table S2. Risk of CKD in the Tiwi population – Stage classification of eGFR and ACR. a) Stage classification using eGFR. b) Stage classification of kidney function using ACR. c) Risk status stratification using the combination of eGFR and ACR using KDIGO 2020 nomenclature. Table S3. The table shows the narrow sense SNP based heritability for the kidney phenotype available in the phenotype data along with its 95% confidence interval and significance level. Significance level less than 0.05 consider to significant heritable trait. Table S4. Factor score loadings for the different phenotypes involved in the factor analysis. The grouped variables are considered for the multiple phenotype GWAS association analysis and are indicated using different colors. Table S5. Allele Frequency in the UKBB population for independent SNPs and Tiwi population. Table S6. Effect size of HbA1c GWAS with identified risk SNPs. Figure S1. Correlation heatmap plot for the available phenotypes in the clinical data. The color was given based on the absolute correlation value. The right-side stacked bar chart represents the factor loading score observed in EFA The x-axis of the plot represents the factor loading scores. [file 13073_2024_1299_MOESM1_ESM.docx]

Additional File 1

Table of Contents

[Table S1: The summary statistics for all the available phenotypes (N = 492) including kidney function, blood pressure, BMI, diabetes, etc. 2](#_Toc157435209)

[Table S2: Risk of CKD in the Tiwi population – Stage classification of eGFR and ACR. a) Stage classification using eGFR. b) Stage classification of kidney function using ACR. c) Risk status stratification using the combination of eGFR and ACR using KDIGO 2020 nomenclature 3](#_Toc157435210)

[Table S3: The table shows the narrow sense SNP based heritability for the kidney phenotype available in the phenotype data along with its 95% confidence interval and significance level. Significance level less than 0.05 consider to significant heritable trait. 4](#_Toc157435211)

[Table S4: Factor score loadings for the different phenotypes involved in the factor analysis. The grouped variables are considered for the multiple phenotype GWAS association analysis and are indicated using different colors. 5](#_Toc157435212)

[Table S5: Allele Frequency in the UKBB population for independent SNPs and Tiwi population. 6](#_Toc157435213)

[Table S6: Effect size of HbA1c GWAS with identified risk SNPs 7](#_Toc157435214)

[Figure S1: Correlation heatmap plot for the available phenotypes in the clinical data. The color was given based on the absolute correlation value. The right-side stacked bar chart represents the factor loading score observed in EFA The x-axis of the plot represents the factor loading scores (Vignesh et al., Figure S1.pdf) 7](#_Toc157435215)

# Table S1: The summary statistics for all the available phenotypes (N = 492) including kidney function, blood pressure, BMI, diabetes, etc.

| *No* | *Quantitative Traits* | *Unit* | *min* | *max* | *Mean (SD)* | *Median (Q1, Q3)* |
| --- | --- | --- | --- | --- | --- | --- |
| *1* | Age | years | 17.00 | 75.00 | 39.55 (12.22) | 39.00 (30.0, 48.00) |
| *2* | Weight | Kg | 35.40 | 145.90 | 67.17 (15.90) | 65.65 (56.77, 75.92) |
| *3* | Height | Cm | 106.00 | 189.00 | 165.00 (8.10) | 165.10 (160.00, 171.00) |
| *4* | Body Mass Index | kg/m^2^ | 13.83 | 55.18 | 24.65 (5.71) | 23.81 (20.54, 27.98) |
| *5* | Waist | cm | 38.00 | 224.00 | 90.20 (16.90) | 88.00 (80.00, 100.00) |
| *6* | Systolic Blood pressure | mm Hg | 76.00 | 188.00 | 113.82 (14.22) | 112.00 (104.00, 122.00) |
| *7* | Diastolic Blood pressure | mm Hg | 43.00 | 114.00 | 73.00 (9.49) | 73.00 (67.00, 79.00) |
| *8* | Urine Creatinine | mmol/L | 0.60 | 72.40 | 11.13 (8.82) | 8.90 (4.60, 15.23) |
| *9* | Serum creatinine | g/dL | 2.00 | 806.00 | 83.76 (77.14) | 72.50 (59.75, 86.00) |
| *10* | Serum albumin | μmol/L | 24.00 | 53.00 | 44.05 (3.94) | 44.00 (42.00, 47.00) |
| *11* | Urine albumin | mg/dL | 0.10 | 650.20 | 27.51 (77.75) | 2.10 (0.60, 13.88) |
| *12* | ACR | mg/mmol | 0.052 | 1755.48 | 34.46 (113.53) | 2.27 (0.66, 19.03) |
| *13* | eGFR | mL/min | 4.99 | 251.15 | 103.58 (24.49) | 108.12 (93.55, 118.20) |
| *14* | Uric Acid | mmol/L | 0.10 | 0.70 | 0.35 (0.09) | 0.35 (0.28, 0.41) |
| *15* | Urine osmolality | mmol/kg | 42.00 | 1197.00 | 537.30 (294.01) | 523.00 (277.00, 767.75) |
| *16* | Hba1c | % | 4.70 | 14.00 | 6.32 (1.58) | 5.80 (5.60, 6.10) |

# Table S2: Risk of CKD in the Tiwi population – Stage classification of eGFR and ACR. a) Stage classification using eGFR. b) Stage classification of kidney function using ACR. c) Risk status stratification using the combination of eGFR and ACR using KDIGO 2020 nomenclature

a) Stage classification using eGFR

| ***eGFR stage*** | ***N (%)*** |
| --- | --- |
| Stage 1 (G1) | 388 (78.86) |
| Stage 2 (G2) | 76 (15.44) |
| Stage 3 (G3a & b) | 20 (4.07) |
| Stage 4 (G4) | 1 (0.20) |
| Stage 5 (G5) | 7 (1.42) |

b) Stage classification of kidney function using ACR

| ***ACR stages*** | ***N (%)*** |
| --- | --- |
| Stage 1 (A1) | 274 (55.69) |
| Stage 2 (A2) | 118 (23.98) |
| Stage 3 (A3) | 100 (20.32) |

c) Risk status stratification using the combination of eGFR and ACR using KDIGO 2020 nomenclature

| ***Risk level*** | ***N (%)*** |
| --- | --- |
| Low risk | 271 (55.08) |
| Moderately increased Risk | 118 (23.98) |
| High Risk | 79 (16.05) |
| Very high Risk | 24 (4.88) |

# Table S3: The table shows the narrow sense SNP based heritability for the kidney phenotype available in the phenotype data along with its 95% confidence interval and significance level. Significance level less than 0.05 consider to significant heritable trait.

| Phenotypes | Heritability | 95% Lower CI | 95 % Upper CI | p |
| --- | --- | --- | --- | --- |
| ACR | 0.526 | 0.343 | 0.708 | 5.32 x 10^-14^ |
| Serum albumin | 0.106 | 0.0 | 0.247 | 2.01 x 10^-2^ |
| Serum creatinine | 0.278 | 0.091 | 0.465 | 2.97 x 10^-4^ |
| eGFR | 0.205 | 0.042 | 0.368 | 8.79 x 10^-4^ |
| Uric acid | 0.357 | 0.169 | 0.544 | 1.13 x 10^-6^ |
| urine albumin | 0.484 | 0.302 | 0.666 | 4.02 x 10^-12^ |
| Urine creatinine | 0.348 | 0.139 | 0.557 | 3.25 x 10^-4^ |
| Urine Osmolality | 0.445 | 0.251 | 0.639 | 1.07 x 10^-7^ |
| Hba1c | 0.117 | 0.0 | 0.272 | 4.36 x 10^-2^ |
| CI – Confidence Interval | | | | |

# Table S4: Factor score loadings for the different phenotypes involved in the factor analysis. The grouped variables are considered for the multiple phenotype GWAS association analysis and are indicated using different colors.

| **Traits** | **Factor 1** | **Factor 2** | **Factor 3** | **Factor 4** | **Factor 5** |
| --- | --- | --- | --- | --- | --- |
| **Weight** | 0.900 |  | 0.113 | 0.176 | 0.368 |
| **Waist** | 0.847 |  |  | 0.104 |  |
| **Serum albumin** | -0.387 | 0.200 | 0.173 |  | 0.182 |
| **BMI** | 0.972 |  | 0.130 | 0.143 |  |
| **Hba1c** | 0.307 | 0.192 | -0.103 |  |  |
| **Urine albumin** | 0.119 | 0.879 |  | 0.113 |  |
| **Serum creatinine** |  | 0.729 |  |  |  |
| **eGFR** | -0.111 | -0.577 |  | -0.254 |  |
| **ACR** | 0.164 | 0.869 |  |  |  |
| **Urine creatinine** | -0.122 |  | 0.968 |  | 0.195 |
| **Urine osmolality** |  |  | 0.765 |  |  |
| **SBP** | 0.103 | 0.178 |  | 0.794 | 0.121 |
| **DBP** |  |  |  | 0.776 |  |
| **Uric acid** | 0.118 | 0.135 | 0.152 | 0.311 | 0.375 |
| **height** |  |  |  |  | 0.989 |
| **SS loadings** | 2.827 | 2.541 | 1.719 | 1.494 | 1.380 |
| **% Variance** | 0.177 | 0.159 | 0.107 | 0.093 | 0.086 |
| ****Loadings =>0.10** | | | | | |
|  | | | | | |

| Table S6: Allele Frequency in the UKBB population for independent SNPs and Tiwi population.  \| Trait \| Chr \| bp \| ID \| UKBB population \| \| \| \| \| \| \| Tiwi \| \| --- \| --- \| --- \| --- \| --- \| --- \| --- \| --- \| --- \| --- \| --- \| --- \| \| African \| British \| Caribbean \| Chinese \| Indian \| Irish \| Pakistan \| \| CGAA_PC_1 \| 4 \| 49304522 \| rs1425534646 \| NA \| NA \| NA \| NA \| NA \| NA \| NA \| 5.07% \| \| 15 \| 37218869 \|  \| NA \| NA \| NA \| NA \| NA \| NA \| NA \| 6.15% \| \| ACR \| 6 \| 154966925 \| rs9689640 \| 96.18% \| 77.42% \| 93.34% \| 65.03% \| 67.33% \| 76.04% \| 69.83% \| 94.70% \| \| 14 \| 100812018 \|  \| NA \| NA \| NA \| NA \| NA \| NA \| NA \| 5.14% \| \| 22 \| 23164355 \| rs11704318 \| 0.00% \| 0.20% \| 0.04% \| 0.78% \| 0.32% \| 0.28% \| 0.42% \| 6.40% \|  Table S7: Effect size of HbA1c GWAS with identified risk SNPs  \| **Phenotype** \| **Risk allele** \| **Alt Allele** \| **Effect Size** \| **Std. error** \| **p** \| \| --- \| --- \| --- \| --- \| --- \| --- \| \| **ACR** \| **rs11704318** \| G \| -0.075 \| 0.130 \| 0.565 \| \| **rs9689640** \| G \| 0.027 \| 0.145 \| 0.850 \| \| **chr14:100812018:T:A** \| A \| 0.084 \| 0.129 \| 0.514 \| \| **CGAA_PC_1** \| **rs1425534646** \| A \| 0.281 \| 0.128 \| 0.028 \| \| **chr15:37218869:A:G** \| G \| 0.245 \| 0.131 \| 0.061 \|  Figure S1: Correlation heatmap plot for the available phenotypes in the clinical data. The color was given based on the absolute correlation value. The right-side stacked bar chart represents the factor loading score observed in EFA The x-axis of the plot represents the factor loading scores.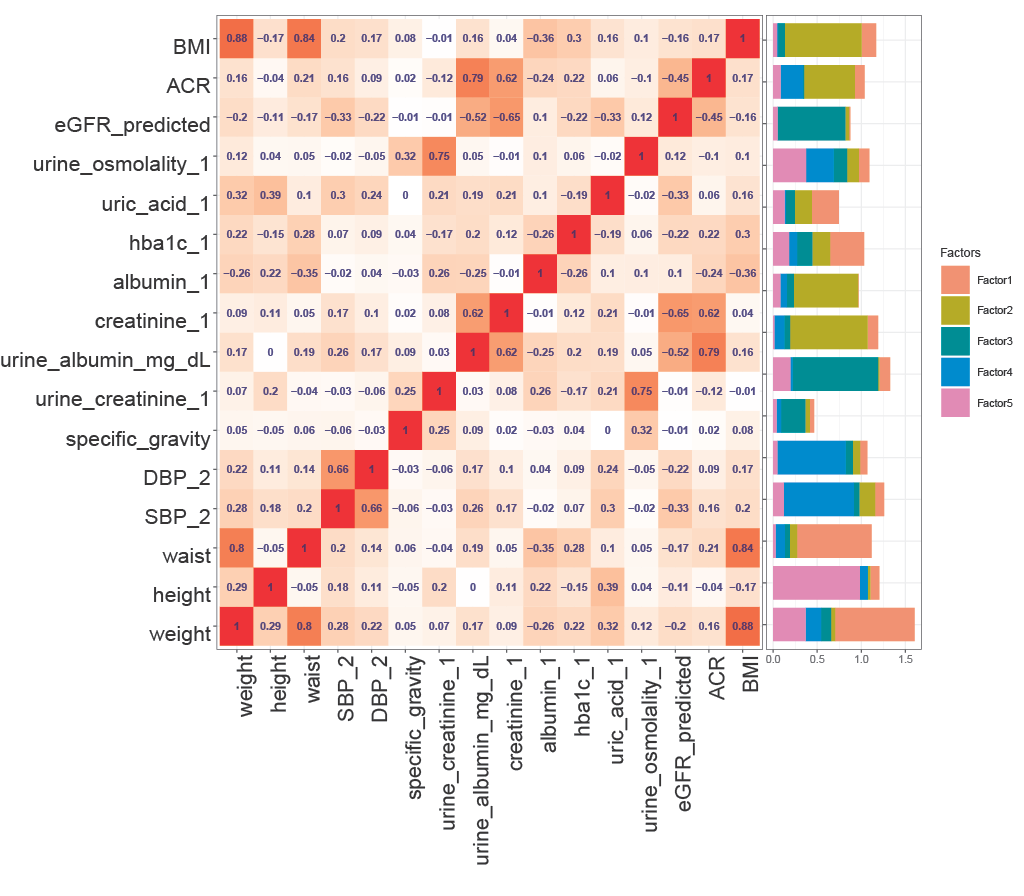 |
| --- | --- | --- | --- | --- | --- | --- | --- | --- | --- | --- | --- | --- | --- | --- | --- | --- | --- | --- | --- | --- | --- | --- | --- | --- | --- | --- | --- | --- | --- | --- | --- | --- | --- | --- | --- | --- | --- | --- | --- | --- | --- | --- | --- | --- | --- | --- | --- | --- | --- | --- | --- | --- | --- | --- | --- | --- | --- | --- | --- | --- | --- | --- | --- | --- | --- | --- | --- | --- | --- | --- | --- | --- | --- | --- | --- | --- | --- | --- | --- | --- | --- | --- | --- | --- | --- | --- | --- | --- | --- | --- | --- | --- | --- | --- | --- | --- | --- | --- | --- | --- | --- | --- | --- | --- | --- | --- | --- | --- | --- |
